# Web-based collaborative model development in interdisciplinary consortia: Design principles and practical guidance

**DOI:** 10.1371/journal.pbio.3003825

**Published:** 2026-06-23

**Authors:** Marvin van Aalst, Alienor Lahlou, Tanvir Hassan, William Gaultier, David Colliaux, Anna Matuszyńska

**Affiliations:** 1 Computational Life Science, Department of Biology, RWTH Aachen University, Aachen, Germany; 2 Sony Computer Science Laboratories - Paris, France; 3 Chimie Physique et Chimie du Vivant, Département de chimie, École normale supérieure, PSL University, Sorbonne Université, CNRS, Paris, France; 4 Center for Computational Life Sciences, RWTH Aachen University, Aachen, Germany

## Abstract

Web-based modelling platforms can enhance collaboration between modelers and experimentalists during early model development. This Community Page provides guiding principles on how to build agile interactive modelling tools.

## Teaser

Web-based modeling platforms can enhance collaboration between modelers and experimentalists during early model development. Drawing on two interdisciplinary case studies, we provide guiding principles on how to build interactive agile modeling tools.

## Introduction

Computational mechanistic models, based for example on ordinary differential equations (ODEs) or flux balance analysis, have become an integral part of how biological hypotheses are formulated, tested, and refined. In parallel, the community has made major progress in making such models FAIR [1], establishing common file formats and standards for describing simulation experiments [2]. Curated repositories such as BioModels [3] have built on this progress to become central public resources for mechanistic models, with strong emphasis on annotation, provenance, and reuse. Alongside this progress, web-based simulation environments, such as JWS Online [4], have enabled reproducible, browser-based execution of mechanistic models. Platforms such as the Cell Collective [5] and BioSimulations [6] extended this ecosystem by enabling interactive re-execution of standardized models without local software installation. Together, these tools define the current baseline of the field: mechanistic models can be shared, curated, and reproducibly simulated through web-based interfaces.

However, most of this infrastructure is optimized for models that are already finished: fully specified, curated, and deposited. In collaborative projects, the most difficult and consequential work often happens before a model reaches that state. During initial and intermediate phases of model development, structure and parametrization remain negotiable, competing hypotheses are explored, and feedback from experimental collaborators can reshape modeling decisions. At this stage, models typically live in private code repositories or notebooks and are shared through static figures, making assumptions difficult to interrogate and feedback slow. This gap is particularly evident in modern biological research, which is increasingly organized around large, interdisciplinary consortia. Substantial public investment is directed toward collaborative programs designed to integrate experimental and theoretical expertise. The promise of this investment is undermined if working models cannot be shared in a form that collaborators can actually test, question, and understand while the modeling is still taking shape.

**Fig 1:**
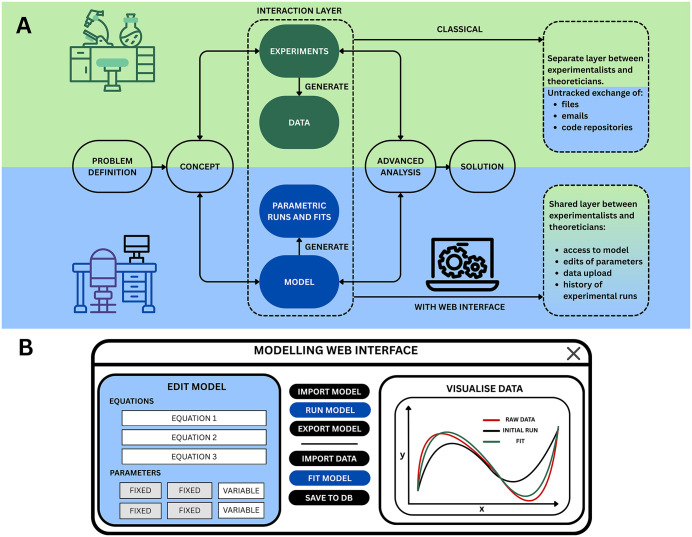
Web-based interaction reshapes how experimental feedback enters the modeling cycle. **A**. The schematic contrasts a classical workflow with a web-enabled collaborative workflow. In the classical setting, experiments generate data that inform modeling and analysis, but interaction between experimentalists and theoreticians occurs through a separate, untracked layer (e.g., files, emails, code repositories), typically late in the process. Introducing a web-based interaction layer integrates models, parametric runs, and fits into a shared space accessible to both communities. This shared layer allows direct access to models, interactive parameter exploration, data upload, and tracking of experimental and modeling runs, thereby placing experimental feedback inside the iterative modeling loop rather than outside it. Interactive exploration enables earlier scrutiny of assumptions, faster feedback, and tighter coupling between experimentation and model refinement within interdisciplinary consortia. **B**. A schematic representation of a web-based modeling interface, illustrating the key interaction points between a user and an evolving model. Models can be imported, exported, and edited directly in the browser. Data can be imported in the interface, and the model can be run with manual adjustment of the parameters or fitted against the data. Minimal visualization of the output is provided. Ideally, all simulation runs are logged into a database to monitor model evolution.

Our experience suggests that the same web technologies that made published models more accessible can be used earlier, during model development, so that models become shared, interactive objects rather than remaining locked in code and scripts. Borrowing from agile software development, where short iterative cycles and continuous stakeholder feedback replace rigid upfront specification, we argue that the same principle applies to modeling, especially with interdisciplinary consortia. Rather than delivering a finished model, teams can expose working prototypes to experimental collaborators at an early stage and revise continuously as new data and insights emerge. Web-based access to evolving model prototypes places experimental partners inside the iterative modeling loop (Fig 1), enabling interactive exploration and immediate feedback without installing software, managing dependencies, or reading code.

In this Community Page, we draw on two case studies, developed independently in Aachen, Germany, and Paris, France, to illustrate how browser-based interaction can accelerate iteration and improve cross-disciplinary scrutiny (Box 1). From these experiences, we derive general design principles and practical guidance for building such platforms, including concrete tool recommendations and deployment strategies, useful both to teams seeking a starting point and to more advanced users.

**Box 1. Case studies of developing web-based modeling platforms**

**Case study 1: mxlweb**

Mxlweb is a general-purpose web application for making ordinary differential equations (ODEs) executable in the browser; here, we used it to simulate the subpopulations of a synthetic community as an illustrative example.

- Context: Collaborative Research Consortium on microbial networking (CRC 1535 – Microbial Networking).
- Collaborating teams: synthetic, micro, and cell biology plus computational modeling.
- Research question: Under what conditions can three species with distinct metabolic strategies coexist in a batch culture?
- Purpose of the web interface: Work-in-progress ODE models were made directly executable in the browser, allowing collaborators to interactively explore parameter ranges, initial conditions, and dynamics.
- Achievement: Direct model access helped partners identify mismatches with experimental intuition at an early point, prioritize new experiments, and tighten the iterative cycle of prediction, experimentation, and model refinement.

**Case study 2: ModelXplorer**

ModelXplorer is a general-purpose web application for fitting ODE-based models; here, we used it to study the photoprotective mechanism nonphotochemical quenching as an illustrative example.

- Context: European project on chlorophyll fluorescence kinetics (DREAM).
- Collaborating teams: Biophysics of photosynthesis, kinetic chemistry plus computational modeling.
- Research question: How to model the fluorescence kinetic signature of stress response in microalgae to high levels of light?
- Purpose of the web interface: The models can be edited in the interface, data can be imported, and models can be simulated or fitted against the data. All runs are logged in a database to track model evolution and enable replay of past configurations.
- Achievement: Systematic logging reduced the risk of losing well-performing configurations, enabling broader exploration of model space. A brick-by-brick modeling approach emerged, in which equation subsets were fitted on targeted experimental conditions (e.g., mutants), allowing parameters to be evaluated separately before assembling the full model.

A more detailed description of both case studies and their technical implementation is available on Zenodo.

## Design principles for agile model development platforms

Rather than sharing figures or code, the models should directly be executable on the user side, allowing collaborators to explore parameter ranges, initial conditions, time scales, and even model architectures. The primary goal of such a modeling platform is to support a tight feedback loop between experimental insight and model structure. Adopting an agile approach to model development requires platforms designed around the same core values: short feedback cycles, transparency, and continuous collaboration. To transform a model from a private computational object into a shared artifact that can be interrogated by all partners, such web-based platforms need to follow several design principles. Taken together, the below principles favor ease of use over analytical completeness. A platform that is actually used by experimentalists, even in a limited way, contributes more to the collaboration than a feature-rich system that remains in the hands of modelers alone.

## Models need to be transparent

Rather than treating models as black boxes, platforms should expose variables, parameters, and equations in a form readable by non-modelers. This can be achieved either through a structured graphical representation, such as an editable graph of operations, encoded, for example, in MathML [7] (see Case study 1, Box 1) or through direct text input of equations (see Case study 2, Box 1). If text input is allowed, inputs must be sanitized and the application sandboxed to avoid security vulnerabilities. A transparent model shifts discussions from simulation outputs toward the underlying mechanistic hypotheses. Once a model reaches a stable and fully specified state, exporting it to SBML for deposition in platforms such as FairdomHub/JWS remains the recommended route for long-term archiving and broader reuse.

## Models need to be immediately responsive

Low-latency calculation and a responsive interface are the two technical requirements for immediate feedback. For models of moderate complexity (a small ODE system with 20 equations), numerical integration can run directly in the browser using a WebAssembly-compiled backend (e.g., Python via Pyodide [8], with scipy.integrate). For heavier computations, a server-side backend is more appropriate. Simulation errors should be communicated with informative and potentially actionable feedback.

## The interface should be accessible without technical barriers

The user interface can be built using a JavaScript framework (e.g., Svelte) or a dashboard library (e.g., Plotly Dash), both of which support reactive, real-time displays. The choice depends on the web development experience available in the team. For browser-based deployments, free hosting on GitHub Pages or Hugging Face Spaces makes the interface accessible through a simple URL. For computationally heavier models, a lightweight Python backend (e.g., Flask or FastAPI) deployed on an institutional server or a cloud service such as Render is sufficient. When data are sensitive, self-hosted deployment behind institutional authentication is preferable. To ensure long-term usage, the platform should be versionable and easy to set up (i.e., via a containerized deployment).

## Platforms need to support asynchronous and iterative use

Platforms should support asynchronous use, allowing partners to revisit and interact with models independently between meetings. Features such as switching between parameter sets or model variants, downloading models and simulation artifacts, and fitting models to imported data can significantly enhance collaboration when clearly integrated into the exploratory workflow. These features typically require a lightweight database backend, such as an SQL database (e.g., PostgreSQL [9]) or a document store (e.g., MongoDB [10]).

## Conclusions

The two case studies (Box 1) show that web-based interaction does not change whether a model is iterated, but where that iteration occurs. Deploying work-in-progress models through the browser placed experimental collaborators directly inside the modeling loop, enabling earlier scrutiny of assumptions, faster feedback, and more exploratory testing. Although developed independently and for different questions, both approaches led to similar outcomes. Lowering the technical barrier increased the flow of experimental feedback and created space for more exploratory testing, ultimately enabling new iterative modeling strategies.

As interdisciplinary consortia increasingly shape biological research, web-native collaborative model development can complement existing standards and repositories by strengthening integration between experimental and theoretical work while models are still taking shape. Both platforms presented here are open source and can be adopted or adapted for other projects. Technical details, deployment instructions, and our Case studies as working examples are provided on Zenodo.

## Abbreviation:

ODEs: ordinary differential equations
